# The perceptions and priorities of professionals in health and social welfare and city planning for creating a healthy living environment: a concept mapping study

**DOI:** 10.1186/s12889-021-11151-7

**Published:** 2021-06-06

**Authors:** Kristine Mourits, Koos van der Velden, Gerard Molleman

**Affiliations:** Department of Primary and Community Care, Radboud University Medical Center, Radboud Institute for Health Sciences, P.O. Box 9101, 6500 HB Nijmegen, The Netherlands

**Keywords:** Concept mapping, Urban health, Policy, City planning, Civil servants

## Abstract

**Background:**

It is helpful for collaboration if professionals from the field of health and social welfare and the field of city planning are aware of each other’s concepts of what a healthy living environment entails and what its components are. This study examined perceptions about creating a healthy living environment of professionals from these two fields, as well as the differences between them.

**Methods:**

We recruited 95 professionals from Nijmegen, the Netherlands who worked in the fields of health, social welfare and city planning in governmental and non-governmental capacities. We used the concept mapping method to collect and analyse their thoughts on healthy living environments. Participants first submitted statements on this subject in a brainstorming session, using an online mapping tool. Then they sorted these statements and rated them on priorities and opportunities within urban planning processes.

**Results:**

During the brainstorm, 43 professionals generated 136 statements. After the elimination of duplicates, 92 statements were individually sorted by 32 professionals. Concept mapping software was used to create an overall map, in which the statements were sorted into ten clusters. Each of these clusters represented one of the main features of a healthy living environments. After 36 participants rated these statements, it emerged that professionals from both fields agreed on priorities and opportunities for the clusters ‘Spatial quality’ and ‘Conducive to exercise’. Professionals also agreed on which three clusters had the fewest priorities and possibilities (‘Promotes personal wellbeing’, ‘Encourages healthy choices’, ‘Conducive to social connections’).

**Conclusion:**

We found that professionals in health and social welfare and city planning have similar views concerning the most and least important features of a healthy living environment in urban planning process. This could indicate that the differences between the two fields may be more nuanced and specific than previously thought. This knowledge offers perspectives for professionals to strengthen their collaboration and to come to a joint result in urban planning projects.

**Supplementary Information:**

The online version contains supplementary material available at 10.1186/s12889-021-11151-7.

## Background

It is generally agreed that health is determined by human biology, lifestyle, health care, and social and physical environments. These factors must be taken into account by Dutch municipalities that formulate policies to protect and promote health. Therefor, policies can use various strategies, like promoting people’s personals skills, strengthening community action and creating supportive environments [1, 2]. This requires linking of activities and cooperation between different policy areas and with different parties in a city’s administration. By law, Dutch municipalities bear significant responsibility for how the living environment is shaped, for instance how traffic is managed, where housing will be built and how much space there will be for greenery. In time, the Netherlands will see the introduction of a new environmental law, of which one objective is a healthy living environment.

To achieve a healthy living environment, the importance of health must be embedded in the process of decision-making for urban planning in our municipalities [3, 4]. Health Impact Assessment (HIA) is one of the approaches which offers government a practical tool that measures the potential impact of policy and urban planning projects on health. However, HIA is rarely used by local governments because of lack of tools, knowledge and interest [5], which means that other approaches are needed to generate attention for health in urban planning processes. For instance, this can be achieved by applying the techniques of ‘integrated health policies’ and ‘health in all policies’, where civil servants within local governments collaborate to incorporate health into other non- health policies, such as urban planning, housing and mobility [6–14].

However, collaboration between health and these different policy fields does not happen naturally. There are several obstacles to the collaboration between the fields of health and social welfare and the city planning. Professionals working in health and social welfare come from the world of welfare, care, education, and health, while professionals in city planning are involved with the world of urban planning, housing, and traffic. These worlds differ in the kind of knowledge they process, their working methods and scope, their political goals and interests, their culture and languages [15–18]. In addition to these differences, conflicting interests between policy objectives do not stimulate cooperation either [12]. Furthermore, most government organizations are structured in such a way that the departments of city planning and of health and social welfare are located at different workplaces. As a result, civil servants do not interact with each other in their daily work [19].

When attempting to bridge the gap between these two different worlds and foster health in urban planning processes, previous research has focused on identifying the differences between the two fields and on increasing knowledge about what constitutes a healthy living environment [3, 14, 15, 20–23]. Nevertheless, successful incorporation of health into urban planning processes requires more detailed investigation of the differences between the worlds of health and social welfare and city planning.

Previous studies have suggested that differences in language contribute to a narrow definition of and narrow perceptions about health. To implement health in all policies, it is important to have a shared concept of what makes a healthy living environment [24, 25]. Urban planning processes often use a narrow definition of health, which is a major barrier in integrating health in urban planning according to several studies [15]. Previous research has focused on identifying differences, but little on what exactly these differences are in perceptions about a healthy living environment, how large are these differences, and are they relevant?

In addition, it is important to examine how different professionals prioritize the elements of a healthy living environment in the current urban planning process, and to determine whether they see the same opportunities for incorporating these elements into future urban planning processes. In the planning phase, where the most important decisions are made about how to shape the environment, it is especially useful to know in which areas the shared interests can be found.

When stakeholders from both sides share a vision about healthy living environments and recognise each other’s similarities and differences in languages, perceptions and priorities an action perspective arises which strengthens their cooperation, and can bridge the gap between these two worlds. To achieve this goal, this study explores the perceptions of civil servants and professionals in the fields of health and social welfare and city planning about a healthy living environment and analysed their differences in content, priorities and perceived opportunities.

## Methods

To systematically assess the perceptions of different participants about a healthy living environment, the study used a concept mapping method. Concept mapping is a structured method to generate ideas from different groups and assemble them into a common framework; the cluster map [26, 27]. The method consists of three steps: 1) brainstorming based on a focus prompt, 2) sorting the statements from the brainstorm, and 3) rating the statements based on two questions.

### Preparation

To initiate a brainstorm and statement generation, a focus prompt is needed. To determine this focus prompt, a pre-test was performed. Ten people, who had a background in research and health but were not participating in this study, were asked to brainstorm on two different focus prompts and were asked if the focus prompt was clear and invited the generation of statements. Responses provided the basis for choosing the final focus prompt. The focus prompt for this study was: “For me, a healthy living environment is an environment where … …” .

### Generation of statements through brainstorm

The participants (*n* = 95) received a personal email with a personal code inviting them to log in to the online concept mapping tool. After providing informed consent, they were asked to fill in as many statements as they wanted, based on the focus prompt. There was a list of statements, so participants could also see the statements other participants had contributed. A reminder was sent after 10 days, and after 20 days the brainstorm was closed.

The brainstorm produced 113 statements. Statements containing more than one statement in a sentence were split in two, while duplicate statements or statements that had no connection with the focus prompt were removed. To keep the list of statements manageable, statements that more or less overlapped were combined. Finally, this resulted in 92 unique statements.

### Sorting and rating the statements

The next step was sorting and rating the 92 remaining statements. All participants (*n* = 95) were invited again to log in with a personal code into the online concept mapping tool and were asked to sort and rate the 92 statements. Participants sorted the statements into sets which, in their opinion, belonged together, and gave every set a name. The participants rated the statements with two questions on a five-point scale: 1) ‘What priority do you think each statement receives in urban planning and urban planning processes?’ (ranging from 1 = no priority to 5 = high priority); 2) ‘How likely is it, in your opinion, that this statement taken into account during the making of urban planning and in urban planning processes?’ (ranging from 1 = not at all to 5 = very). A reminder was sent after 14 days, and after 33 days the sorting and rating was closed.

### Participants

We began the recruitment process by discussing what professional occupations were most relevant to urban planning processes. On the basis of this discussion, we decided to recruit individuals from Nijmegen who were involved in some way in urban planning developments, either in the field of health and social welfare or in city planning. These people should have a position as politician, manager, policy advisor, process or project manager or executive. Thanks to the network of author K.M., who herself was an employee of the municipality of Nijmegen, we had a clear overview of appropriate candidates and were able to approach a substantial number of all those individuals in Nijmegen who were involved with this subject. The study invited 95 participants to use the online concept mapping system. The distribution of participants was 61 from inside the municipality organization and 34 from outside.

### Analyses

First, the data was checked for completeness. Then, Concept Systems Global MAX software [28] was used for the data analyses. A similarity matrix was conducted, which indicated the number of people who placed a statement in the same set. Based on this matrix and nonmetric multidimensional scaling, a point map was created which showed the relationships between statements. Using this point map we followed the hierarchical cluster analysis procedure of Kane and Trochim [26] to determine the final cluster map. The researchers made the choice to stop at a certain number of clusters on the basis of substantive relevance and distinctiveness of the clusters. The final names of the clusters were selected as follow. Three persons (authors K.M. and G.M. and external party P.P.) independently proposed a name for each cluster based on the statements in that cluster. These proposals were mutually discussed and the final name of the cluster was chosen by consensus [26]. The software calculated a stress value to indicate whether the two-dimensional cluster map of the statements gave a good representation of the input matrix data [26]. The desired range for this value was between 0.21 and 0.37. Furthermore, the software specified a bridging value for each statement, and also for each cluster. This indicated whether statements were frequently sorted with other statements that were nearby (low bridging value), or more often sorted with statements that were further away (higher bridging value).

To analyse whether there were differences between participants working in the field of health and social welfare and in the field of city planning, separate cluster maps were created in the same way as described above [26, 29]. Pattern matches [26] were used to compare responses to the rating questions from the fields of health and social welfare and city planning. The sequence order of the outcome of the pattern matches was used and will be presented in a table. The outcome was presented and discussed in a feedback session with a group of civil servants of the city of Nijmegen.

## Results

### Participants

As shown in Table 1, the response rate for the brainstorming was 45% (*n* = 43), the sorting 34% (*n* = 32) and the rating 38% (*n* = 36). Respondents to the sorting and rating tasks were almost equally divided between men and women and between health and social welfare and city planning. As the sorting task was time-consuming (45 min), some participants did not complete it, or chose to do only the rating task. As a result, the number of participants in rating was higher than in sorting. Twenty-seven participants completed all the tasks (the brainstorm, sorting and rating). We tried to involve politicians in the study, but the response was limited (*n* = 3) and confined to the brainstorming phase. Their contribution was therefore not a sufficient basis for scientific conclusions about their perceptions and priorities. Despite the absence of politicians, the group of responders contained a good mix of professionals from health and social welfare and city planning with a variety of functions.

**Table 1:** Demographics of the respondents who participated in

Categories	Brainstorming (*n* = 43)		Sorting (*n* = 32)		Rating (*n* = 36)	
Response	45%		34%		38%	
**Male/ Female**	N	%	N	%	N	%
Male	22	51.2	19	59.4	20	55.6
Female	21	48.8	13	40.6	16	44.4
**Function**						
Policy	19	44.2	16	50.0	19	52.8
Process	16	37.2	12	37.5	13	36.1
Practice	8	18.6	4	12.5	4	11.1
**Domain**						
Health and social welfare	25	58.1	15	46.9	19	52.8
Male	9	36.0	8	53.3	9	47.4
Female	16	64.0	7	46.7	10	52.6
City planning	18	41.9	17	53.1	17	47.2
Male	11	61.1	11	64.7	11	64.7
Female	7	38.9	6	35.3	6	35.3
**Affinity with the subject**^a^						
None / little			3	9.4	3	8.3
Reasonable			14	43.8	17	47.2
Considerable			15	46.9	16	44.4

^a^ The background information about affinity with the topic is not available from the participants of the brainstorming phase

### Cluster map

**Fig. 1:**
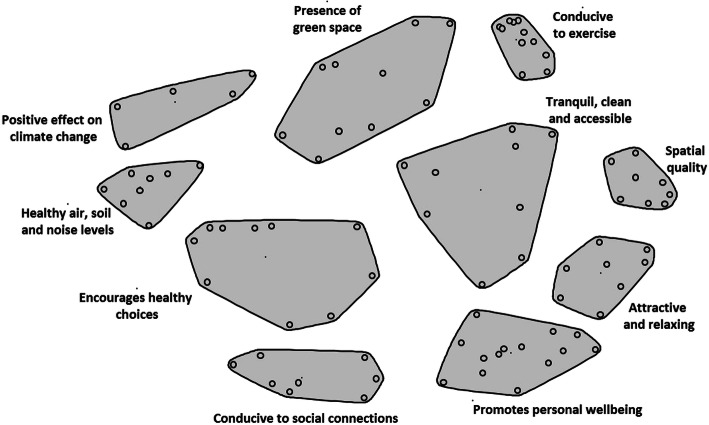
Final concept map healthy living environment in 10 clusters with 92 statements (dots)

All 92 statements were sorted by the participants in clusters ranging in number from 3 to 14. The final cluster map of the data of all participants included ten clusters and is presented in Fig. 1. The appearance of the map and the shape of the clusters arise from the distance between statements (the dots in Fig. 1). A shorter distance between statements indicates that the participants more often sorted those statements together. The stress value of this final cluster map is 0.29. This indicates that there is little discrepancy between the input of the matrix data and the representation of those data on the two-dimensional array [26]. The bridging value of the clusters is between 0.15 and 0.74, which means that some clusters represent a more coherent topic, while other clusters represent a broader concept. The bridging value is an indicator of how ‘anchored’ or frequently sorted the statements are with statements around them. Higher bridging values indicate that the statement is more related to other statements in other clusters, but they say nothing about the validity of the statement [23].

Using the naming method described earlier, the following names were chosen for the ten clusters: ‘Spatial quality’, ‘Attractive and relaxing’, ‘Tranquil, clean and accessible’, ‘Promotes personal wellbeing’, ‘Conducive to exercise’, ‘Presence of green space’, ‘Positive effect on climate change’, ‘Healthy air, soil and noise levels’, ‘Encourages healthy choices’, and ‘Conducive to social connections’.

To identify possible differences between participants in the health and social welfare group and the city planning group, a separate cluster map was created for each group. The cluster map for the first of these groups had eight clusters and the cluster map for the second group had ten. Table 2 provides an overview of the three cluster maps with the cluster names, the number of statements within the cluster and the bridging value for each cluster.

**Table 2:** Differences in clusters between cluster map total, city planning and health and social welfare

Total	City Planning	Health and social welfare
Conducive to exercise (11 statements, B = 0.15)	Conducive to exercise (15 statements, B = 0.17)	Conducive to exercise (15 statements, B = 0.33)
Promotes personal wellbeing (14 statements, B = 0.23)	Wellbeing for everyone (12 statements, B = 0.29)	Personal wellbeing (9 statements, B = 0.59)
Tranquil, clean and accessible (9 statements, B = 0.45)	Tranquillity (7 statements, B = 0.17)	Clean, without hindrances (10 statements, B = 0.49)
Healthy air, soil and noise levels (8 statements, B = 0.60)	Good climate and environment (7 statements, B = 0.61)	
Positive effect on climate change (5 statements, B = 0.74)		Green and not vulnerable to climate change (7 statements, B = 0.49)
Presence of green space (10 statements, B = 0.48)	Nature and green spaces (5 statements, B = 0.78)	
Attractive and relaxing (8 statements, B = 0.47)	Pleasant (6 statements, B = 0.40)	
	Public spaces and facilities (11 statements, B = 0.50)	Diversity and accessibility (9 statements, B = 0.50)
Conducive to social connections (9 statements, B = 0.49)	Social connections (11 statements, B = 0.81)	Pleasant to live alongside one another (18 statements, B = 0.28)
Spatial quality (8 statements, B = 0.60)	Attractive (7 statements, B = 0.62)	High quality and safe (15 statements, B = 0.62)
Encourages healthy choices (10 statements, B = 0.60)	Encourages healthy choices (11 statements, B = 0.43)	Healthy facilities (9 statements, B = 0.77)

B = mean bridging value for clusters between 0 and 1. The lower the bridging value, the more coherence between the statements within the cluster. The higher the bridging value, the more statements in the cluster also match statements in other clusters

The Appendices present the cluster maps for health and social welfare and city planning, as well as a table with the clusters and the statements in each cluster for all three cluster maps.

### Priorities and opportunities in the clusters

Table 3 presents the results of the two rating questions, which are based on the final cluster map of all participants. The first three columns show the outcome of the pattern matches about priority in urban planning process for all participants, for city planning and for health and social welfare, in descending order. The last three columns show the outcome of the opportunities for the cluster in urban planning process for all participants, for the city planning and for health and social welfare, in descending order. All participants gave ‘Spatial quality’ and ‘Conducive to exercise’ the most priority in urban development processes, while ‘Conducive to social connection’ and ‘Promotes personal wellbeing’ received the least priority. Participants also saw the most opportunities for ‘Spatial quality’ and ‘Conducive to exercise’ in urban development processes, and they saw the fewest opportunities for ‘Encourages healthy choices’ and ‘Promotes personal wellbeing’.

**Table 3:** Descending order of clusters for priority and opportunity of the total, city planning, health and social welfare

Total Priority	City planning Priority	Health and social welfare Priority	Total Opportunity	City planning Opportunity	Health and social welfare Opportunity
Spatial quality	Spatial quality	Spatial quality	Spatial quality	Spatial quality	Spatial quality
Conducive to exercise	Conducive to exercise	Conducive to exercise	Conducive to exercise	Conducive to exercise	Conducive to exercise
Tranquil, clean and accessible	Tranquil, clean and accessible	Attractive and relaxing	Positive effect on climate change	Positive effect on climate change	Presence of green space
Attractive and relaxing	Positive effect on climate change	Tranquil, clean and accessible	Presence of green space	Presence of green space	Tranquil, clean and accessible
Positive effect on climate change	Healthy air, soil and noise levels	Presence of green space	Tranquil, clean and accessible	Tranquil, clean and accessible	Positive effect on climate change
Presence of green space	Conducive to social connections	Positive effect on climate change	Healthy air, soil and noise levels	Healthy air, soil and noise levels	Healthy air, soil and noise levels
Healthy air, soil and noise levels	Attractive and relaxing	Healthy air, soil and noise levels	Attractive and relaxing	Conducive to social connections	Attractive and relaxing
Encourages healthy choices	Encourages healthy choices	Encourages healthy choices	Conducive to social connections	Encourages healthy choices	Conducive to social connections
Conducive to social connections	Presence of green space	Conducive to social connections	Encourages healthy choices	Attractive and relaxing	Encourages healthy choices
Promotes personal wellbeing	Promotes personal wellbeing	Promotes personal wellbeing	Promotes personal wellbeing	Promotes personal wellbeing	Promotes personal wellbeing

The fields of health and social welfare and city planning were in agreement with regard to the clusters ‘Spatial quality’ and ‘Conducive to exercise’. Both domains gave priority to these clusters in the current planning process and saw high opportunities for these two clusters in future urban planning process. There was also great agreement about the clusters ‘Promotes personal wellbeing’ and ‘Encourages healthy choices’, both of which had less priority in current urban planning processes and offered fewer opportunities in the future. There were slight differences between the two fields regarding the priorities of the other clusters.

## Discussion

This study aimed to compare the perceptions of representatives from the fields of health and social welfare and city planning on what constitutes a healthy living environment. We created a cluster map and asked about priorities and opportunities to pinpoint where the participants’ perceptions agreed or diverged. Previous studies [15–17, 24] have suggested clear differences between health and social welfare and city planning. However, we found that these differences in perceptions and interpretation of healthy living environments are more nuanced and specific than previously assumed, and that the two fields agreed on which clusters had the most, or the fewest, priorities and opportunities in urban planning processes.

The fields of health and social welfare and city planning are in agreement about their highest priorities in urban planning processes: ‘Spatial quality’ and ‘Conducive to exercise’. It is striking that the city planning participants assigned a relatively low priority in current planning processes to the cluster ‘Presence of green space’, especially since the city of Nijmegen was European Green Capital in 2018 [30]. It would be interesting to do further research on this result and find an explanation for it. We focused our study on professionals who are directly involved in local urban planning, because their priorities are of course a major determinant of which matters receive the most attention in urban planning processes. However, it would also be good to include those matters that residents find important in the process. In a survey about healthy living environments residents ranked the ten clusters of the overall cluster map according to their importance [31], and indicated the following top three priorities: 1) ‘Healthy air, soil and noise levels’; 2) ‘Tranquil, clean and accessible’; and 3) ‘Presence of green space’. This difference between professionals and residents should be examined more closely in further research, because agreement or shared priorities is important in an urban planning process.

Both health and social welfare and city planning saw the most opportunities in urban planning processes for ‘Spatial quality’ and ‘Conducive to exercise’. The fact that all participants saw few opportunities for the cluster ‘Encourages healthy choices’ is interesting, because there is growing attention in research and public health [32, 33] for nudging and the theory that a person’s behaviour can be changed through minor interventions in the social or urban environment [34]. This could possibly indicate that civil servants and professionals are not yet sufficiently familiar with nudging and its possibilities. From the perspective of current practice, the low position of the cluster ‘Conducive to social connections’ is also notable. Participants thought that it offered few opportunities in the urban planning process. This is interesting because Den Broeder et al. [35] shows that health professionals see strengthening the social infrastructure in a neighborhood as a possible method of improving the health of the inhabitants, and Kent et al. [25] suggested that community interaction is one of the three domains where urban planning can most effectively support health and well-being. Furthermore, an important priority in many municipalities is strengthening and improving social contacts in neighbourhoods by creating parks and community gardens [36], as this contributes to quality of life, social cohesion and prevention of loneliness. In order to inspire professionals and civil servants to recognize the opportunities in this cluster and the possible uses of nudging, it could be helpful to organize scrum sessions, inspirational meetings or organisational workshops.

We certainly see differences between the fields of health and social welfare and city planning, but these are much more specific and less generic than previously thought. The cluster map of the field of health and social welfare shows eight clusters, while the cluster map of the field of city planning has ten. The difference of two clusters may indicate that professionals from the city planning make more distinction in clusters. The health and social welfare professionals merge more statements into one cluster, for example ‘High quality and safe’ and ‘Clean, without hindrance’ and ‘Pleasant to live alongside one another’, while the city planning professionals assign fewer statements to each cluster, which produces a higher number of clusters, including ‘Nature and green spaces’ and ‘Tranquility’. This may suggest that the health and social welfare professionals thinks about healthy living environments from a broader perspective, and that the city planning professionals see more specific elements. Our study shows that, even though health and social welfare feels that ‘Green and not vulnerable to climate change’ and ‘Clean, without hindrances’ do belong together, these are different topics according to the city planning professionals. When professionals from both groups are in conversation, they need to be aware of these differences in order to understand each other. This idea was also supported during the feedback session with the civil servants of Nijmegen. They were in agreement with the results and indicated that an important follow-up could involve finding shared interpretations of what the clusters mean. For example, conversation partners should mutually specify at the start of a project what the content of a cluster is. This is in line with the findings of Lge-Elegbede et al. [18] concerning to the importance of a shared understanding of different perspectives, which is important for collaboration and effective decision-making in urban planning processes.

It should be emphasised that this study was done in a specific context and that the participants and author K.M. are part of the same network, which may have had an effect on the results. However, since we carefully considered which occupations were most relevant to the study and asked the open brainstorm question: “For me, a healthy living environment is an environment where … …” , we believe that the bias remained limited. Other limitations of this research are the low response of politicians and the lack of underlying motivation or reasons behind statements and sorting statements. In order to sound out the politicians’ perspective on a healthy living environment, a different approach is needed. Background information about choices could be very valuable, as it might promote mutual communication and understanding. Nonetheless, the clusters that are mentioned in the cluster map are reflected in the literature [3, 20, 21] as important elements for a healthy living environment, and this study shows that there is more agreement on many points than previously thought. This is a good starting point for further collaboration to incorporate health in urban planning processes [37], as shared views can significantly strengthen a collaboration.

## Conclusions

We have found that professionals in the fields health and social welfare and city planning have a consistent view on the most and least important features of a healthy living environment in urban planning processes, and that the differences between the two fields are more nuanced and specific then previously assumed. This knowledge can help professionals to strengthen their collaboration and come to a joint result in urban planning projects. However, collaboration alone is not enough to tackle a wicked problem [38] like health issue, and there are other differences that need to be addressed. In future research, it is also important to consider how to deal with differences in interests, scope, and working processes.

## Supplementary Information

**Additional file 1: Fig. A.** Final cluster map for city planning.

**Additional file 2: Fig. B.** Final cluster map for health and social welfare.

**Additional file 3: Table A.** Overview of cluster names and their statements of all three cluster maps.

## Data Availability

The data underlying this article are available in the article and in its online supplementary material. Further data underlying this article cannot be shared publicly due to the privacy of individuals that participated in the study. The data will be shared on reasonable request to the corresponding author.

## Abbreviation

HIA: Health Impact Assessment
