# Supplementary figures and images for: The perceptions and priorities of professionals in health and social welfare and city planning for creating a healthy living environment: a concept mapping study
Source: BMC Public Health. 2021 Jun 6;21:1085. doi: 10.1186/s12889-021-11151-7 (PMC8180117; doi:10.1186/s12889-021-11151-7)

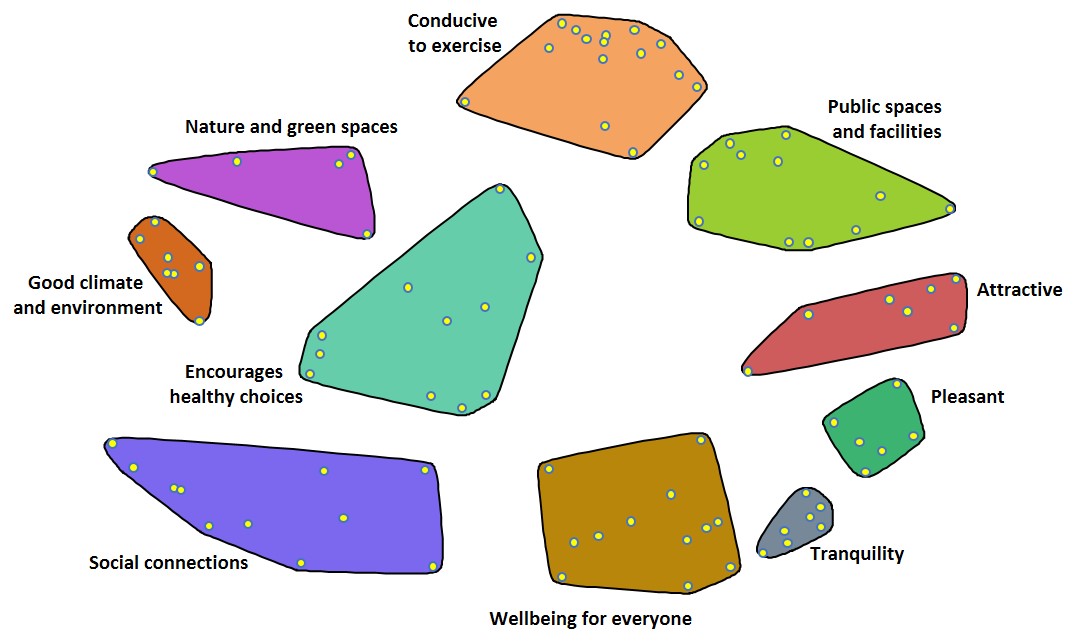

Supplement: Supplementary file 1 — Additional file 1: Fig. A. Final cluster map for city planning. [file 12889_2021_11151_MOESM1_ESM.jpg]

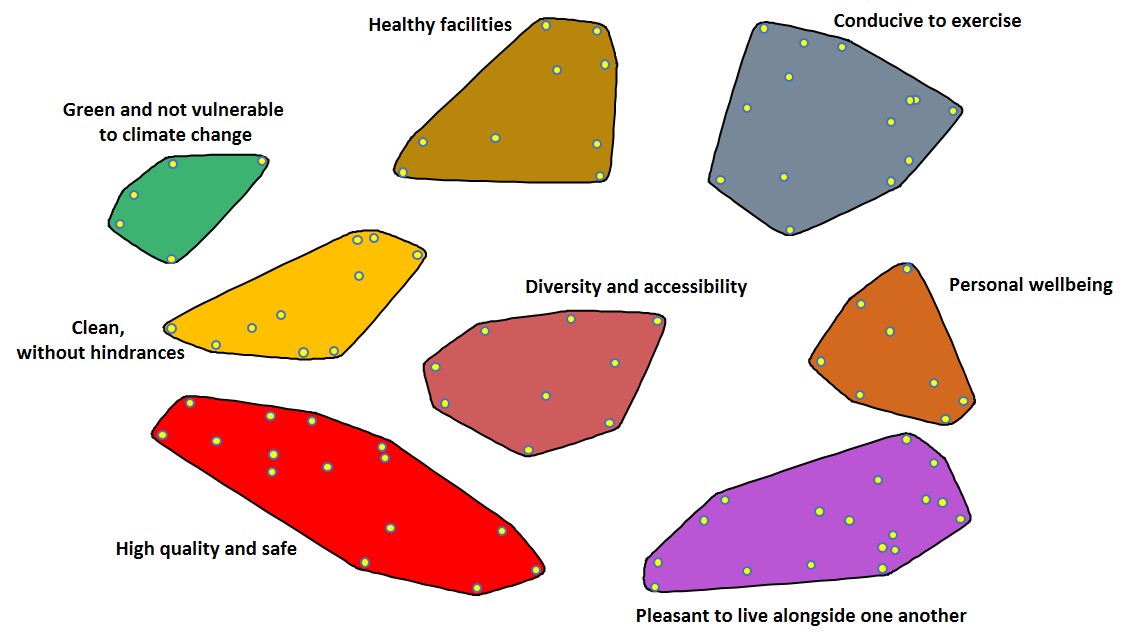

Supplement: Supplementary file 2 — Additional file 2: Fig. B. Final cluster map for health and social welfare. [file 12889_2021_11151_MOESM2_ESM.jpg]
