# Supplementary material for: The perceptions and priorities of professionals in health and social welfare and city planning for creating a healthy living environment: a concept mapping study
Source: BMC Public Health. 2021 Jun 6;21:1085. doi: 10.1186/s12889-021-11151-7 (PMC8180117; doi:10.1186/s12889-021-11151-7)
Supplement: Supplementary file 3 — Additional file 3: Table A. Overview of cluster names and their statements of all three cluster maps. [file 12889_2021_11151_MOESM3_ESM.docx]

Supplementary material to article “The perceptions and priorities of professionals in health and social welfare and city planning for creating a healthy living environment: a concept mapping study”

Kristine Mourits, Koos van der Velden, Gerard Molleman

**Table A Cluster name and statements of all three cluster maps**

| **Cluster name**  **All participants** | Statements | **Cluster name**  **City planning** | Statements | **Cluster name Health and social welfare** | Statements |
| --- | --- | --- | --- | --- | --- |
| **Spatial quality**  **(8 statements)**  **B = 0.60** | visually attractive environment with beautiful buildings, art, greenery  few high-rise buildings because this feels oppressive  high-quality landscaping  has a good mix of functions and activities  sufficiently supplied with suitable housing  sufficient facilities available (shops, sports clubs, community centres, GPs, district nursing care)  feels spacious  makes you want to stay there | **Attractive**  **(7 statements)**  **B = 0.62** | visually attractive environment with beautiful buildings, art, greenery  high-quality landscaping  smells goog  few high-rise buildings because this feels oppressive  for all people who live there - regardless of age, diversity, health and lifestyle - offers a good mix between peace and green, social encounter, proximity to basic services  feels spacious  makes you want to stay there | **High quality and safe**  **(15 statements)**  **B = 0.62** | Provided with a good infrastructure  visually attractive environment with beautiful buildings, art, greenery  has low parking availability  is whole (not broken)  sufficient facilities available (shops, sports clubs, community centres, GPs, district nursing care)  high-quality landscaping  few high-rise buildings because this feels oppressive  safe traffic regulation  air, soil, and noise levels are safe  the right balance between periods with and without noise  a place where there is sufficient social control  is arranged in cooperation within the possible (legal and administrative) limits  is safe, for young and old, during the day or in the evening  sufficiently supplied with suitable housing  is open to change |
| **Attractive and relaxing**  **(8 statements)**  **B = 0.47** | pleasant to live in  provides space for finding peace  open (not limited, restricted)  provides space for relaxation  is whole (not broken)  surprising surroundings  has a good mix of ‘liveliness’ and ‘livability’  conducive to relaxation | **Pleasant**  **( 6 statements)**  **B = 0.40** | pleasant to live in  open (not limited, restricted)  surprising surroundings  privacy offers.  is whole (not broken)  has a good mix of ‘liveliness’ and ‘livability’ | **Diversity and accessibility**  **(9 statements)**  **B = 0.50** | open (not limited, restricted)  surprising surroundings  makes you want to stay there  has a good mix of functions and activities  has a good mix of ‘liveliness’ and ‘livability’  not over-stimulating  environment with quiet places  that poses no obstacles for the elderly and people with a functional disability (handicap). This way they can move around without impediments  for all people who live there - regardless of age, diversity, health and lifestyle - offers a good mix between peace and green, social encounter, proximity to basic services |
| **Tranquil, clean and accessible**  **(9 statements)**  **B = 0.45** | not over-stimulating  offers privacy  clean (no dog or cat poo)  clean (in terms of rubbish)  has low parking availability  environment with quiet places  provided with a good infrastructure  that poses no obstacles for the elderly and people with a functional disability (handicap). This way they can move around without impediments.  for all people who live there, regardless of age, diversity, health and lifestyle - offers a good mix between peace and greenery, social encounters, proximity to basic services | **Public spaces and facilities**  **(11 statements)**  **B = 0.50** | lawns and open spaces  provided with a good infrastructure  decorative greenery  a healthy setting with room for your own garden  has low parking availability  offers space for children to fool around: secret corners, wasteland, not just ‘planned' playgrounds and play areas sufficiently supplied with suitable housing  clean (in terms of rubbish)  clean (no dog or cat poo)  sufficient facilities available (shops, sports clubs, community centres, GPs, district nursing care)  has a good mix of functions and activities | **Clean, without hindrances**  **(10 statements)**  **B = 0.49** | clean (in terms of rubbish)  is free from dust pollution  clean (no dog or cat poo)  is free from stench  is free of noise  facilitates car use as little as possible  is free from air pollution  smells good  feels spacious  privacy offers. |
| **Promotes personal wellbeing**  **(14 statements)**  **B = 0.23** | where you feel comfortable  provides space for creating your own happiness  makes it possible to become or be who I am  spatial and social aspects make me feel good: both physically and mentally, whether indoors or outdoors  contributes to well-being  feels like home  a place in which you feel comfortable.  gives a sense of physical, mental and cognitive well-being  makes me relax mentally  is cosy  allows people to make their own choices  is good for mind, body and nature; gives a physical, mental and cognitive feeling of wellbeing, contributes to wellbeing, enjoyment in living, feeling healthy and experiencing a general sense of meaning  a pleasant working atmosphere radiates from the people who work there  is open to change | **Tranquillity**  **(7 statements)**  **B = 0.17** | feels like home  spatial and social aspects make me feel good: both physically and mentally, whether indoors or outdoors  makes me relax mentally  where you feel comfortable  conducive to relaxation  is open to change  provides space for finding peace | **Green and not vulnerable to climate change**  **(7 statements)**  **B = 0.49** | lawns and open spaces  A healthy environment has room for public green areas  decorative greenery  can mitigate the negative effects of climate change  promotes biodiversity  has mainly real nature sounds such as birdsong and rustling trees instead of car, train and construction sounds  if public space or private garden is sufficiently green to combat heat stress |
| **Conducive to exercise**  **(11 statements)**  **B = 0.15** | conducive to physical recreation such as cycling, walking, running, sailing  conducive to walking  conducive to cycling  inspires you to use walking, running or cycling paths to make the link between home and park, or home and nature reserve  conducive to exercise  encourages you to discover how beautiful the city is as you exercise  a setting where young people are encouraged to use the basic forms of movement  conducive to playing  has walking routes for the elderly and rest areas/ benches  makes it easy to play, move and exercise in the immediate vicinity of the home  offers space for children to fool around: secret corners, wasteland, not just ‘planned' playgrounds and play areas | **Good Climate and environment**  **(7 statements)**  **B = 0.61** | Is free from dust pollution  is free from stench  discourages smoking  is free from air pollution  is free of noise  can mitigate the negative effects of climate change  air, soil, and noise levels are safe | **Healthy facilities**  **(9 statements)**  **B = 0.77** | discourages unhealthy choices  has no fast food chains.  contains a lot of water suitable for exercise  movement  discourages smoking  offers space for children to fool around: secret corners, wasteland, not just ‘planned' playgrounds and play areas  healthy cooking, both in institutional kitchens and at home  a healthy setting with room for your own garden  contains lots of greenery that encourages exercise  provides space for discovering nature |
| **Presence of green spaces**  **(10 statements)**  **B = 0.48** | contains a lot of water suitable for exercise  contains lots of greenery that encourages exercise  facilitates car use as little as possible  a healthy setting with room for your own garden  has no fast food chains  lawns and open spaces  provides space for discovering nature  decorative greenery  safe traffic regulation  offers sufficient opportunities to behave healthily in various ways | **Nature and green spaces**  **(5 statements)**  **B = 0.78** | if public space or private garden is sufficiently green to combat heat stress  A healthy environment has room for public green areas  is safe, for young and old, during the day or in the evening  has mainly real nature sounds such as birdsong and rustling trees instead of car, train and construction sounds  promotes biodiversity | **Conducive to exercise**  **(15 statements)**  **B = 0.33** | conducive to cycling  conducive to exercise  has walking routes for the elderly and rest areas/ benches  encourages you to discover how beautiful the city is as you exercise  conducive to playing  conducive to walking  conducive to physical recreation such as cycling, walking, running, sailing  a setting where young people are encouraged to use the basic forms of movement  inspires you to use walking, running or cycling paths to make the link between home and park, or home and nature reserve  stresses the importance of health  encourages the making of healthy choices  sufficient examples of healthy behaviour  offers sufficient opportunities to behave healthily in various ways  makes it easy to play, move and exercise in the immediate vicinity of the home  conducive to relaxation |
| **Positive effect on climate change**  **(5 statements)**  **B = 0.74** | if public space or private garden is sufficiently green to combat heat stress  a healthy environment has room for public green spaces  can mitigate the negative effects of climate change  has mainly real nature sounds such as birdsong and rustling trees instead of car, train and construction sounds  promotes biodiversity | **Encourages Healthy choices**  **(11 statements)**  **B = 0.43** | discourages unhealthy choices  encourages the making of healthy choices  sufficient examples of healthy behaviour  the right balance between periods with and without noise  has no fast food chains.  healthy cooking, both in institutional kitchens and at home  stresses the importance of health  does not negatively affect my health  environment with quiet places  offers sufficient opportunities to behave healthily in various ways  safe traffic regulation | **Pleasant to live alongside one another**  **(18 statements)**  **B = 0.28** | enables people to live in harmony with each other  where you have a social network to fall back on  is cosy  stimulates meetings between all target groups  where you feel comfortable  contributes to well-being  produces pleasant social contacts  feels like home  in which you feel comfortable.  conducive to social interaction  stimulates encounter  makes it possible to become or be who I am  gives room to live together happily, to work, to relax and to live in consideration for each other  a pleasant working atmosphere radiates from the people who work there  allows people to make their own choices  recognizes and appreciates diversity  pleasant to live in  offers security for development, growth and participation |
| **Healthy air, soil and noise levels**  **( 8 statements)**  **B = 0.60** | is free from stench  is free of noise  is free from dust pollution  healthy cooking, both in institutional kitchens and at home  free from air pollution  discourages smoking  stresses the importance of health  air, soil, and noise levels are safe | **Conducive to exercise**  **( 15 statements)**  **B = 0.17** | inspires you to use walking, running or cycling paths to make the link between home and park, or home and nature reserve  conducive to cycling  conducive to physical recreation such as cycling, walking, running, sailing  contains a lot of water suitable for exercise  a setting where young people are encouraged to use the basic forms of movement  conducive to walking  conducive to exercise  contains lots of greenery that encourages exercise  encourages you to discover how beautiful the city is as you exercise  conducive to playing  makes it easy to play, move and exercise in the immediate vicinity of the home  has walking routes for the elderly and rest areas/ benches  provides space for discovering nature  that poses no obstacles for the elderly and people with a functional disability (handicap). This way they can move around without impediments  facilitates car use as little as possible | **Personal wellbeing**  **(9 statements)**  **B = 0.59** | provides space for creating your own happiness  spatial and social aspects make me feel good: both physically and mentally, whether indoors or outdoors  is good for mind, body and nature; gives a physical, mental and cognitive feeling of wellbeing, contributes to wellbeing, enjoyment in living, feeling healthy and experiencing a general sense of meaning  gives a sense of physical, mental and cognitive well-being  provides space for finding peace  makes me relax mentally  an environment that is healthy for everyone’s body and soul, no matter how diverse  provides space for relaxation  does not negatively affect my health |
| **Encourages healthy choices**  **(10 statements)**  **B = 0.60** | the right balance between periods with and without noise  discourages unhealthy choices  encourages the making of healthy choices  an environment that is healthy for everyone’s body and soul, no matter how diverse  smells good  sufficient examples of healthy behaviour  does not negatively affect my health  offers security for development, growth and participation  is arranged in cooperation within the possible (legal and administrative) limits  is safe, for young and old, during the day or in the evening | **Social connections**  **(11 statements)**  **B = 0.81** | offers security for development, growth and participation  enables people to live in harmony with each other  conducive to social interaction  recognizes and appreciates diversity  produces pleasant social contacts  where you have a social network to fall back on  a place where there is sufficient social control  gives room to live together happily, to work, to relax and to live in consideration for each other  is arranged in cooperation within the possible (legal and administrative) limits  stimulates meetings between all target groups  stimulates encounter |  |  |
| **Conducive to social connections**  **(9 statements)**  **B = 0.49** | enables people to live in harmony with each other  where you have a social network to fall back on  produces pleasant social contacts  conducive to social interaction  gives room to live together happily, to work, to relax and to live in consideration for each other  recognizes and appreciates diversity  stimulates meetings between all target groups  a place where there is sufficient social control  stimulates encounter | **Wellbeing for everyone**  **(12 statements)**  **B = 0.29** | Where you feel comfortable  gives a sense of physical, mental and cognitive well-being  contributes to well-being  provides space for creating your own happiness  makes it possible to become or be who I am  is good for mind, body and nature; gives a physical, mental and cognitive feeling of wellbeing, contributes to wellbeing, enjoyment in living, feeling healthy and experiencing a general sense of meaning  not over-stimulating  provides space for relaxation  allows people to make their own choices  an environment that is healthy for everyone’s body and soul, no matter how diverse  is cosy  a pleasant working atmosphere radiates from the people who work there |  |  |
| **B=mean bridging value for clusters between 0 and 1** | | | | | |
